# Decreasing In-home Smoking of Adults—Results from a School-based Intervention Program in Viet Nam

**DOI:** 10.3934/publichealth.2016.4.863

**Published:** 2016-10-24

**Authors:** Le Thi Thanh Huong, Tran Khanh Long, Le Vu Anh, Margaret Cook, Mike Capra

**Affiliations:** 1 Hanoi University of Public Health, 1A Duc Thang Road, Duc Thang Ward, North Tu Liem District, Hanoi, Viet Nam; 2 Vietnam Public Health Association; 3 University of Queensland, Australia

**Keywords:** in-home smoking, exposure, secondhand smoke, school-based intervention, children, adults, Viet Nam

## Abstract

It is indicated that children are involuntarily exposed to secondhand smoke from adults, mainly at their home environment. This study aimed at describing the effectiveness of the school-based intervention to decrease the in-home smoking situation of adults so as to decrease children's exposure to secondhand smoke at home during the year 2011–2012 in a rural district in Hanoi, Viet Nam. This school-based intervention program (intervention and control group) involved 804 children aged 8 to 11 years from August 2011 to May 2012 in a rural district of Hanoi, Viet Nam. Children were taught in class about the harmful effects of secondhand smoke and about how to negotiate with fathers not to smoke in-home. Then children applied what they learnt, including staying away from secondhand smoke and persuading fathers not to smoke in-home in order to decrease children's exposure to secondhand smoke. Chi square test, t-test and multinominal logistic regression were applied in data analysis. The results showed that children's reported their father's in-home smoking decreased from 83.0% pre-intervention to 59.8% post-intervention (*p* < 0.001) in the intervention school while no change happened in the control school. The study found that the better changed smoking location of adult smokers as reported by children associated with the school who received intervention activities (adjusted OR = 2.04; 95% CI: 1.28–3.24). Poorer changed attitudes towards secondhand smoke of children associated with a lower percentage of better change in smoking location of their fathers/other adult smokers (aOR = 0.51, 95% CI: 0.28–0.96). Children's poorer changed knowledge towards secondhand smoke also associated with poorer changed smoking location of adult smokers (aOR = 2.88, 95% CI: 1.07–7.76). It is recommended by this study that similar school based intervention approaches should be applied in primary schools in Viet Nam to increase children's awareness on the adverse health effects of secondhand smoke and to help them to be able to avoid their exposure to secondhand smoke at their home environment.

## 1. Introduction

Secondhand smoke (SHS) or environmental tobacco smoke is composed of sidestream smoke—the smoke from a burning cigarette or other burning tobacco products and mainstream smoke from smokers' exhalation [1],[2]. It is a source of widespread excess morbidity and mortality, imposing significant costs on non-smokers and society as a whole [1],[3].

Children's exposure to secondhand smoke (SHS) is a well-known public health problem, with many children at particular risk from adults' smoking. Children are exposed to SHS involuntarily and often face detrimental health effects, including pneumonia and bronchitis, lung function deficit, coughing and wheezing, worsening of asthma, middle ear disease, and sudden infant death syndrome [1],[3],[4]. Their main exposure environment is at home [3].

The exposure of children to SHS in Viet Nam varies depending on the age of children. While children under five years of age exposed to SHS at home was around 70% [5],[6]; the prevalence of children at primary school age was lower and ranged from approximately 38.3% [7] to 52.4% [8]. The proportion of children living with smokers in Viet Nam is also reported to be high, at around 60% [7] or 66% [8] among children 8 to 11 years of age and at 70% among children under five years of age [5]. Several intervention studies in different provinces showed that the exposure of children to SHS decreased significantly after the intervention, however, the prevalence still remained high at around 50% [9],[10]. In a study in Thai Binh province in 2009, the prevalence of smokers smoked inside their houses was very high (97%) and 87% smokers reported to smoke when children were around [11]. In another study with children at primary school age, children who lived with smokers reported that the prevalence of smokers smoked inside their houses was 78.5% [12].

International interventions with children showed that, if children learn and understand a new health behavior at school, they tend to apply that behavior in their daily life without enforcement from health programmers or other adults [13]. Furthermore, children can also communicate the health behaviors to other people, including their parents, their siblings, their peers and communities [14],[15]. However, this is not the case for younger children. For example, children less than 8 years old are still limited in language development [16]. It is also difficult for these younger children to stay focused on an issue for a period of time, and therefore they can easily lose interest [16]. Children from 8 to 11 years of age, on the other hand, have longer attention spans, and therefore can help better in delivering an intervention program to adults [16]. An intervention study with primary school children in the north of Viet Nam showed that children at the age of 6 could maintain healthy behaviors themselves, but were restricted in their ability to communicate healthy behaviors to their siblings and their parents, while children at the ages of 8 and 9 or older could communicate healthy behaviors to other members of their families [17]. Children in Viet Nam, especially at primary school age, may be influential in stopping their parents' smoking in their presence [18], and parents in Vietnam tend to listen to, learn from and share opinions with their children on different aspects of living [17].

This article aimed at describing the effectiveness of the school-based intervention to decrease the in-home smoking situation of adults so as to decrease children's exposure to SHS at home during the year 2011–2012 in a rural district in Hanoi, Viet Nam.

## 2. Materials and Methods

### 2.1. Trial design

This intervention was a quasi-experimental design, with an intervention and a control group.

### 2.2. Sites and Assignment of the Intervention and the Control Group

The study was conducted in Chuong My district, a rural district of Hanoi, Viet Nam where there had been no previous survey of tobacco use and no previous tobacco intervention program. The selection of communes in the district was based on the following criteria:

- not in the two towns of the district
- not adjacent to the two towns of the district
- having similar socio-demographic-economic characteristics
- not having the national roads running through the communes
- not having boundaries with other districts/provinces
- having only one primary school in the commune, and a similar number of classes per school and a similar number of school children
- having children who spend only half a day at school.

After the selection, two communes met these above criteria: Quang Bi and Trung Hoa. Then from the two selected communes, the study chose two primary schools in the two communes (Quang Bi Primary School and Trung Hoa Primary School). The two primary schools were randomly assigned as the intervention group (children at Quang Bi Primary School) and the control group (children at Trung Hoa Primary School).

### 2.3. Study Participants and Sample Size

The study recruited children at primary school age from 8 to 11 years old. All children aged 8 to 11 (who were studying at grade 3 through grade 5) in the two selected primary schools were the study participants. Totally, 804 children participated in the study (397 children in the intervention school and 407 in the control school). This sample size was large enough to meet the requirement of the sample size for this intervention study, as indicated by Huong's study (2014) [12]. Among the intervention group, all children received intervention activities in classroom settings. Only those who lived with current smokers applied what they learnt in class into their daily activities to prevent their exposure to SHS at home.

### 2.4. Timeline of the Study

The timeline of this study was from August 2011 to May 2012. The pre-intervention survey was undertaken in August 2011(quantitative data completed by primary school children with self-administered questionnaire, SAQ). The intervention was implemented from November 2011 to April 2012. The post-intervention survey was conducted in May 2012 (SAQ filled by children).

### 2.5. Study Protocol

The study was conducted in three phases: pre-intervention survey, intervention, and post-intervention survey. In phase 1, all children in Grade 3 to Grade 5 in the two selected primary schools were invited to participate in the pre-intervention survey. In the classroom setting, with teachers' presence, self-administered questionnaires were distributed to all children for whom consent had been obtained. The teachers helped distribute the questionnaire to their pupils and asked their pupils to fill in the form. Written instructions on how to fill in the form were attached to the questionnaire to avoid teachers' instruction bias as much as possible. In parallel, audio-instructions were played in class, so that all the participating children could hear the same voice in the same tone instructing them how to fill in the questionnaire. The content and delivery of the audio-instructions were based on results of the pilot study using children from grades 3, 4 and 5. All teachers who participated in the surveys undertook a workshop on the questionnaire that was facilitated by the principal investigator. These teachers were able to help clarify the content/meaning of the questions and also clarify any difficulties in understanding the audio-instructions. The data collection tool included the self-administered questionnaire filled by the recruited children, which included questions on their personal information, smoking places of adult smoker(s) if they lived with smoker(s), attitude and practices of children against SHS. Attitudes of children toward SHS included 9 questions about children's feelings when they saw their fathers or other adult smokers living in their family smoked in-home or in their vicinity, and their confidence in successfully persuading their fathers or other adult smokers not to smoke inside their homes. Practices of children included: (1) persuading adults not to smoke in-home and/or (2) going away when there was SHS nearby them.

Phase 2: During the intervention from November 2011 to April 2012, children in the intervention school were involved in classroom teaching sessions, mainly cartoon and simple texts about the concept of SHS, the harmful effects of SHS to children's health, what attitudes children should have when being exposed to SHS, and the practices they should follow in the circumstance of being exposure to SHS. In addition to that, they were also involved in series of role plays in class, where they practiced how to negotiate with adults on appropriate smoking places. A drawing competition on harmful effects of SHS and how to persuade adult smokers to smoke outside the house was organized in March 2011 with the voluntarily participation of children in the intervention school. After two weeks, 225 pictures were collected. On 26 March 2011, a competition on “Understanding the harmful effects of SHS to children's health” was organized. Each class from grade 3 to grade 5 chose one team to participate in the competition. The awards were given to the three best pictures (selected by the school committee) and the three best teams in the competition on the date of 26 March 2011. At home environment, children who lived with smokers were required to stay away from secondhand smoke and to persuade their smoking fathers or other smoking adults living in the same house with them not to smoke in the home [12].

In phase 3 at post-intervention, data collection was undertaken similar to pre-intervention.

### 2.6. Variables of the Study

Knowledge of children on SHS harmful effects on health was assessed by 5 questions. Each correct answer was given 1 mark. The highest score was 9. Then children's knowledge was assessed in three levels: poor (0 to 2), fair (3 to 4) and good (5 to 9). Range of good knowledge level was broader than the other two levels because children were required to list diseases caused by SHS exposure, each correct disease was given 1 mark. Similarly, attitudes of children towards SHS were assessed by 9 questions (what children felt when then saw someone smoke, what they felt when they saw their fathers smoke, their confidence in convincing adults not to smoke in the home). Each correct attitude was scored with 1 mark. Poor attitude ranged from 0 to 3, fair attitude ranged from 4 to 6 and good attitude ranged from 7 to 9. Lastly, children's practices were assessed by 5 questions to ask what children did at home when they saw someone smoked or their fathers/other adult smokers in their home smoked. Each correct answer received 1 mark. Children's practices score ranged from 0 to 5 and was categorized as poor (0 to 2), fair (3 to 4) and good (5).

The dependent variable was the change of smoking place of children's parents or other smokers in children's families. This variable was defined based on the adults' smoking pattern with respect to smoking indoors/outdoors before and after the intervention.

The independent variables included:

- Intervention (Yes/No)
- Age of children
- Gender (boys, girls)
- Number of siblings
- Change in children's knowledge post- vs. pre-intervention on SHS and its harmful effects to children's health (Table 1).
- Change in children's attitudes post- vs. pre-intervention on avoiding exposure to SHS (Table 1).
- Change in children's practices post- vs. pre-intervention on avoiding exposure to SHS (Table 1).

**Table 1.** Variables of knowledge, attitudes and practices of children regarding the SHS and the change in smoking location of children's parents/other smokers in their family.

Variable	Value	Classification criteria	
		Pre-intervention	Post-Intervention
The change in smoking location of children's fathers/smokers in children's family	1. poorer changed	Smoking outdoor	Smoking indoor
	2. Unchanged	Smoking indoor	Smoking indoor
	3. Better changed	Smoking indoor/outdoor	Smoking outdoor
The change in children's knowledge/attitude/practices post vs. pre intervention	1. poorer changed	Good	Fair/poor
		Fair	poor
	2. Unchanged	Good/fair/poor	Good/fair/poor
	3. Better changed	Poor	Fair/good
		Fair	Good

### 2.7. Data Management and Analysis

Data was cleaned and entered through the use of the Epidata version 3.0. Data was analyzed by the SPSS IBM software 19.0. Descriptive statistics were used to present the characteristics of participants. Chi square test, t-test were applied to identify the difference between the intervention and the control groups. Crude Odd ratio of independent variables was calculated based on Cochran's and Mantel-Haenszel test. The association of dependent variable and all independent variables such as age, gender, intervention, number of sibling, change of knowledge and attitude were tested using Chi-square test. The independent variables which had associated with dependent variable with *p* < 0.2 were entered in the multinominal logistic regression to calculate the adjusted OR of independent variables and the change in smoking behavior of the adults within the children's home. The collinearity between factors in the model was tested using VIF.

### 2.8. Ethical Considerations

Ethical approval of this study was granted by both the Hanoi School of Public Health and the University of Queensland Behavioral and Social Sciences Ethical Review Committee (ethical clearances numbers 008/2011/YTCC-HD3 dated 10 March 2011 and 2011000250 dated 25 March 2011 respectively).

## 3. Results

There were totally 804 children to be recruited in the study in both the intervention and the control school. The general information about the recruited children is presented in Table 2. It is revealed from the Table that there were neither significant differences regarding gender, age and grade of children nor the status of children living with smokers in the family, with approximately two thirds of children reported to live with smokers in their families in both schools. However, there was significant different regarding the average number of siblings and the average number of smokers living in the same house with children. No significant difference was seen among those who were the smokers in children's family in the two schools. Among those who lived with smokers, more than 80% reported that their fathers smoked, followed by uncles and grand-fathers as smokers. Only a few children reported to live with smoking mothers/aunts/grand-mothers.

**Table 2.** General information about children in the intervention and the control schools.

Characteristics of the studied children		Intervention School (n = 397)		Control School (n = 407)		*p* value
		n	%	N	%	
Gender	Boy	201	51.0	213	52.0	0.692*
	Girl	196	49.0	194	48.0	
Grade	3	114	29.0	124	30.0	0.518*
	4	141	36.0	129	32.0	
	5	142	36.0	154	38.0	
Living with smokers	Yes	264	66.5	266	65.4	0.733*
	No	133	33.5	141	34.6
Age (Mean ± SE)		9.1 ± 0.40		9.1 ± 0.40		0.980^#^
Number of sibling (Mean ± SE)		1.5 ± 0.50		1.8 ± 0.50		<0.0001^#^
Number of smokers (Mean ± SE)		1.3 ± 0.04		1.2 ± 0.03		0.035^#^
Smokers	Father	213	80.7	216	81.2	0.879*
	Brother	14	5.3	15	5.6	0.865*
	Grand-father	51	19.3	38	14.3	0.121*
	Uncle	54	20.5	46	17.3	0.352*
	Others (mother, grand-mother, aunt, sister)	5	1.3	7	1.7	0.590*

* Chi square test; ^#^ t-test.

Results on children's knowledge on SHS harmful effects to children's health and children's attitudes and practices towards SHS at pre- and post-intervention time are presented in Table 3. There was a sharp increase in children's knowledge (good level) in the intervention school, and an increased trend in good knowledge also was found in the control school at post-intervention time, but the prevalence of children with good knowledge at post-intervention time was significantly higher than that of the control school (*p* < 0.001). Similar trend was found with regards to the attitudes and practices of children towards SHS.

**Table 3.** Children's knowledge, attitudes and practices on SHS and their fathers'/adult smokers' smoking location.

		Pre-intervention					Post-intervention				
		Intervention School		Control School		p*	Intervention School		Control School		p*
		**n**	**%**	**n**	**%**		**n**	**%**	**n**	**%**	
Knowledge on SHS harmful effects to children's health	poor	273	68.8	297	73.0	**0.10**	10	2.5	185	45.5	**<0.001**
	fair	111	28.0	105	25.8		64	16.1	131	32.2	
	good	13	3.3	5	1.2		323	81.4	91	22.4	
	**Total**	**397**	**100**	**407**	**100**		**397**	**100**	**407**	**100**	
Attitudes towards SHS	poor	87	21.9	78	19.2	**<0.001**	2	0.5	43	10.6	**<0.001**
	fair	157	39.5	216	53.1		133	33.5	204	50.1	
	good	153	38.5	113	27.8		262	66.0	160	39.3	
	**Total**	**397**	**100**	**407**	**100**		**397**	**100**	**407**	**100**	
Practices towards SHS	poor	132	50.0	178	66.9	**<0.001**	5	1.9	138	51.9	**<0.001**
	fair	106	40.2	74	27.8		122	46.2	91	34.2	
	good	26	9.8	14	5.3		137	51.9	37	13.9	
	**Total**	**264**	**100**	**266**	**100**		**264**	**100**	**266**	**100**	
Smoking location of fathers/adult smokers	In-home	219	83.0	200	75.2	**0.03**	158	59.8	202	75.9	**<0.001**
	Outdoor	45	17.0	66	24.8		106	40.2	64	24.1	
	**Total**	**264**	**100**	**266**	**100**		**264**	**100**	**266**	**100**	

* Chi square test

**Figure 1.**
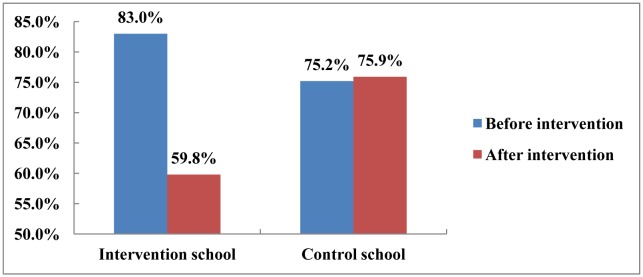
The percentage of fathers/adult smokers smoking in the home pre- and post-intervention, as reported by children who lived with smokers.

Children, who reported to live with smokers, were asked to report the smoking location of their fathers/other adult smokers (in-home or outdoors). The results of the smoking place of children's fathers or other smokers at pre- and post-intervention surveys are represented in Figure 1 and Table 3.

As indicated in Table 3 and Figure 1, among children who reported to live with smokers, at pre-intervention, the prevalence of children reporting that adult smokers smoked in-home was significantly higher in the intervention school than those in the control school (83.0% versus 75.2%, *p* = 0.028). Post-intervention, the proportion of fathers and others smoking in the home in the intervention school was significantly lower than that in the control school (59.8% versus 75.9%, *p* < 0.001). The decrease in the proportion of adult smokers smoking in the home post-intervention in the intervention school was significant (59.8% vs 83.0%; χ^2^ = 34.512, *df* = 1, *p* < 0.001). In contrast, at the control school, the percentage of fathers and others smoking post-intervention (75.9%) was unchanged as compared to pre-intervention (75.2% versus 75.9%) (χ^2^ = 0.041, *df* = 1, *p* = 0.840).

In order to describe factors associated with the change in smoking place of adult smokers living in the same house with children, multinominal logistic regression was applied. Results are presented in Table 4.

According to Table 4, in the school that received intervention, children reported an adjusted odd ratio of 2.04 times higher of better change of smoking place of their fathers (versus unchanged) as compared to fathers of children in the control school (95% CI: 1.28–3.24). In relation to children's attitude towards SHS, children with poorer changed attitude reported a lower percentage of 49% of better change of their fathers'/other adults' smoking place than those with remained unchanged practice with a crude odd ratio of 0.51 (95% CI: 0.28–0.96). Regarding knowledge of children towards SHS, children with poorer changed knowledge reported higher poorer change of smoking place of their fathers/other adults in their families (cOR = 2.58, 95% CI: 1.00–6.64; aOR = 2.88, 95% CI: 1.07–7.76).

Other factors such as age, gender, number of siblings, better changes in practice of children towards SHS did not associate with better changed nor poorer changed smoking location of fathers or adult smokers living in the same house with children.

**Table 4.** Associated factors with the change of smoking location of fathers /other adult smokers living in the same house with primary school children (n = 530).

	Poorer changed vs. unchanged		Better changed vs. unchanged	
	cOR^#^ (95% CI)	aOR* (95% CI)	cOR^#^ (95% CI)	aOR* (95% CI)
**Age**	0.94 (0.69–1.28)		1 (0.7–1.41)	
**Number of siblings**	0.90 (0.69–1.17)		1.07 (0.8–1.45)	
**Gender**				
Male	0.86 (0.51–1.45)	0.83 (0.48–1.43)	0.76 (0.50–1.14)	0.8 (0.54–1.18)
Female	1	1	1	1
**Intervention**				
Yes	0.55 (0.31–0.95)	0.54 (0.28–1.04)	2.16 (1.41–3.31)	**2.04 (1.28–3.24)**
No	1	1	1	1
**Change in attitude**				
Poorer changed	0.99 (0.48–2.05)	0.86 (0.41–1.82)	0.59 (0.30–1.17)	**0.51 (0.28–0.96)**
Better changed	0.90 (0.50–1.62)	0.87 (0.47–1.59)	1.36 (0.88–2.12)	1.03 (0.67–1.56)
Remained unchanged	1	1	1	1
**Change in practice**				
Poorer changed	0.77 (0.25–2.38)	0.7 (0.22–2.26)	0.98 (0.40–2.42)	0.87 (0.38–2.01)
Better changed	0.87 (0.51–1.50)	1.15 (0.63–2.12)	1.56 (1.02–2.40)	1.16 (0.75–1.79)
Remained unchanged	1	1	1	1
**Change in knowledge**				
Poorer changed	2.58 (1.00–6.64)	**2.88 (1.07–7.76)**	0.52 (0.18–1.50)	0.82 (0.33–2.04)
Better changed	1.10 (0.56–2.15)	1.32 (0.65–2.69)	0.90 (0.56–1.46)	0.64 (0.39–1.04)
Remained unchanged	1	1	1	1

The reference category is unchange of smoking place of fathers /other adult smokers living in the same house with primary school children. **cOR^#^:** crude odd ratio; aOR*: adjusted odd ratio

## 4. Discussion

It is reported by children involved in the study that around two-thirds of them lived in the same house with smoker(s). This prevalence was relevant to the previous studies in the same age group of children [7], and was a little bit lower than that among younger children in Viet Nam [5],[6]. It could be understood that children at younger age normally spent more time at home than children at the primary school age, and therefore the risk of being exposure to SHS among the older age group might be lower than the younger ages. It was indicated that the majority of smokers in this study was male (and around four-fifths of them were children's fathers). This prevalence was much similar with the results found in the national study in 2010, which indicated that 47.4% of adult males smoked while the percentage among adult females was only 1.4% [19].

Pre-intervention, a total of 83% of children who lived with smokers in the intervention school and 75.2% of children who lived with smokers in the control school reported that their fathers or other adult smokers smoked in the home (Figure 1). This constituted an overall figure of 52.1% of all 804 recruited children living with in-home smokers. This percentage was lower than that found by a study in central Vietnam, where 63.5% children under 5 years of age lived with “indoor smokers” [5]. This could be explained by the fact that children at the primary school age spent their time at both school and at home, while children under five years of age spent more time at home, and this could be the reason why the exposure prevalence to SHS in the under five year group is often reported to be higher than older children. The current study also indicated that the overall proportion of children living with in-home smokers was lower than that found by another study in the north of Vietnam, where 97% of smokers reported frequently smoking in the home, and 87% admitted recurrent smoking in children's vicinity [11].

The study results showed that children's knowledge on the harmful effects of SHS on their health, and their attitudes and practices on avoiding SHS exposure improved significantly in both schools. However, the better improvement was observed among children in the intervention school. Of the children in this school, 81.4% reached the ‘good’ level of knowledge on SHS, while this percentage in the control school was 22.4%. In the Vietnamese primary education curriculum, the content on tobacco control and the harmful effects of SHS is restricted to only two sections of two lessons, one in the Grade 3 subject Nature and Society [20] and one in the Grade 5 subject Sciences [21]. This inclusion could explain the better knowledge on SHS of children at the control school, although this school did not receive any intervention activities from the program. However, it is suggested that the greater improvement in children's knowledge at the full intervention school (Table 3) means that the SHS intervention program significantly contributed to the advancement of those children's knowledge. Many other intervention studies in various areas of public health also revealed that the participating children had attained significantly better knowledge at the conclusion of the interventions [15],[22]–[25]. Similarly to the trend found in the improvement of children's knowledge on SHS and its harmful effects to children's health, the children's attitudes and practices towards SHS was also higher in the intervention school as compared to the control school. The improvement in children's attitudes could be partly due to the teaching of curriculum content about tobacco in Grade 3 [20] and in Grade 5 [21] as mentioned in the previous section. This connection is evidenced in the results of the pilot study by Huong et al. [7] in the same district, with almost all children who participated showing their unhappy feelings when they saw their fathers or anyone else smoking in the home. However, the improvement in attitudes among children in the intervention school was much better than that of their counterparts in the control school. Apart from being exposed to the same teaching content related to tobacco control in the primary education program in Grade 3 and Grade 5, children in the full intervention school were being taught about SHS for the whole 6 months. Again, it might have indicated that the intervention could be a factor that contributed to the better attitudes of children in this school compared to the control school. The marked improvement in children's practices in the intervention school implied that the intervention program played an important role in assisting children to achieve better practices compared to the control school. Other intervention programs in many fields of public health also showed a convincing improvement in children's practices after the intervention, such as in hygiene and diarrhoea prevention in Indonesia [22], dengue prevention in Puerto Rico [25], water and environmental sanitation in Nigeria [24], hygiene, malaria and diarrhoea in Kenya [15], hygiene and hand-washing in China [26], and hand-washing with soap in Vietnam [17].

Post-intervention, the prevalence of fathers/adult smokers who frequently smoked in the home, as reported by children at the full intervention school, decreased significantly (from 83.0% to 59.8%), while almost no difference occurred in the control school. This result was again confirmed by the multinominal logistic regression model (Table 4), with 2.04 times higher of better change in smoking location of fathers or other adult smokers in children's family in the intervention school compared to those in the control school. This impressive result supported the initial achievement of the trial intervention program, with many of the in-home smokers in the intervention school having changed their smoking behavior to smoke outdoors, and therefore contributed to the decrease in children's exposure to SHS at home. Similar results were also obtained in different ‘smoke-free home’ interventions applying similar approaches of teaching about SHS at primary schools in Portugal [27], Pakistan [28], England [29], and Spain [30].

Normally, interventions might have shown results that good knowledge is associated with good attitudes and practices of the study participants, such as hand washing with soap [17] or dengue fever prevention [31]. This study found a result that poorer change in knowledge of children towards SHS was associated with poorer change in smoking location of children's fathers/other adult smokers but better change in children's knowledge did not necessarily lead to better change in smoking location of adult smokers who lived in the same house with children (Table 4). However, it might be explained that better change in smoking location of adult smokers was not arisen from the studied children but from their smoking fathers/other adult smokers. This study also indicated that children with poorer changed attitudes reported a lower odds ratio of better changed smoking location among their fathers or other adult smokers who lived in the same house with them (Table 4). Although the study results did not show that children with better changed attitudes were associated with better change in smoking location of their fathers/other adult smokers, similar results were found in another study in Viet Nam that smokers often paid attention to their children's attitudes when they started burning a cigarette [18].

This current study, although gaining preliminary successful results, did contain some limitations. Firstly, the study was conducted in only one rural district of Hanoi (Chuong My district) and this district was neither representative of Hanoi City nor representative of all rural areas in Viet Nam. The district was chosen because it had no previously known intervention on tobacco control. The selection of the two communes was based on criteria set by the intervention protocol. Secondly, the study could only collect data from primary school children through a self-administered questionnaire (SAQ) in their classroom setting. Due to a shortage of financial and human resources, no questionnaires were completed by parents, and nor were any observations made of the occurrence of smoking and in-home smoking in the locality. Hence, the background information provided by the children, and information on living with smokers and the exposure of children to SHS at home as well as the smoking locations of their fathers or other smokers sharing the house with them were not confirmed by their parents or by observational checklists. Due to children's limited understanding, some variables regarding children's parents, such as parental age, occupations and educational levels, children's family's economic status etc. which might have associated with the smoking location of adults could not be collected through the children. Last but not least, the study targeted primary school children aged 8 to 11. These young children might have misunderstood the content of the SAQ and provided some incorrect or biased responses. However, the study had foreseen this situation and had made preparations to overcome and minimize this challenge. The authors of this study referred to many materials on designing appropriate questions for children at primary school to inform the design of the SAQ [32]–[34]. The SAQ, after being designed, had been pretested in the pilot study in November 2010 [7]. Modifications and adjustments were made according to the results of the pilot study. Before completing the questionnaire in the classroom setting, children were given audio-taped instructions to avoid any bias arising from explanations given by different teachers.

## 5. Conclusions and Recommendations

To conclude, this study confirmed that by end of the intervention, the prevalence of in-home smoking by fathers and other adults living in the same house with children decreased significantly whereas almost no change occurred with children in the control school. It is also indicated by the study results that the intervention program might have contributed to the decreased prevalence of in-home smoking of adult smokers and increased children's awareness on the adverse health effects of SHS and therefore could help to reduce their exposure to SHS in their home environment. It is suggested that similar school-based intervention approaches with primary school children should be applied to a larger scale in Viet Nam, with consideration of all factors such as demographic factors of children's parents that might have related to the parents' smoking habit and their smoking location, and with parents' involvement as interviewees or with observation of smoking habits at home to ensure higher reliability of the intervention's results.
